# Immune-enhancing activity of *C. militaris* fermented with *Pediococcus pentosaceus* (GRC-ON89A) in CY-induced immunosuppressed model

**DOI:** 10.1186/s12906-018-2133-9

**Published:** 2018-02-23

**Authors:** Ha-Kyoung Kwon, Woo-Ri Jo, Hye-Jin Park

**Affiliations:** Department of Food Science and Biotechnology, Gachon University, Sungnam, Gyeonggi-do 461-701 South Korea

**Keywords:** *Cordyceps militaris*, Probiotic fermentation, Immune enhancing, *Pediococcus pentosaceus*, Cyclophosphamide-induced immune suppression, macrophage

## Abstract

**Background:**

*Cordyceps militaris (C. militaris)* is reported to exert various immune-activities. To enhance its activity, we fermented *C.militaris* with *Pediococcus pentosaceus* ON89A (GRC-ON89A). In this study, we investigated the immune-enhancing activity GRC-ON89A, using immunosuppressed model.

**Methods:**

Immunosuppression was induced by intraperitoneal injection of cyclophosphamide (CY). Each group was orally administered distilled water, GRC-ON89A or GRC, respectively. The phagocytic activities against IgG -opsonized FITC particles were measured using phagocytosis assay kit. The contents β-glucan, cordycepin and SCFA were measured using β-glucan kit, liquid chromatography-mass spectrometry analysis and Gas chromatography-mass spectrometry analysis, respectively.

**Results:**

Among GRC fermented with different probiotic strains (*Pediococcus pentossaceus* ON89A, *Lactobacillus pentosus* SC64, *Weissella cibaria* Sal.Cla22), GRC-ON89A induced the highest elevation of nitric oxide production and enhanced phagocytic activity of RAW 264.7 cells. In primary cultured murine macrophages from normal and CY-treated mice, GRC-ON89A increased phagocytic activity, compared to that in control cells. GRC-ON89A also significantly induced the mRNA expression of TNF-α and IL-10 and the levels of phosphorylated Lyn, Syk and MAPK. The contents of β-glucan, cordycepin and SCFA in GRC significantly increased after ON89A fermentation, compared to those in unfermented GRC.

**Conclusion:**

These results indicate that GRC-ON89A exerted the enhanced immunostimulatory activity and contained more nutritional components, compared to unfermented GRC. Our results suggested that GRC-ON89A may be applied as an agent for immune boosting therapy in immune suppressed patients.

## Background

The dysregulation of immune responses and systems aggravates malignancies and infectious diseases of immune surveillance, including microbial detection and host defense mechanisms [1]. Immunosuppression is a state of temporary or permanent immune dysfunction, and can increase organism vulnerability to pathogens. Immune dysfunction is caused by various factors, including aging and chemotherapeutic drugs. Cyclophosphamide (CY) is a cytotoxic alkylating drug, used as a chemotherapeutic drug in childhood and adult malignancies [2]. Many cancer patients with lymphomas and leukemia were primarily treated with CY [3, 4]. However, severe side effects were reported since the CY uptake into normal cells occurs more than in cancer cells [2]. CY induces apoptotic cell death and causes the pronounced cytotoxic effect on lymphocytes [5]. It suppresses the immune system by killing immune cells and suppressing the proliferation and phagocytic activity and Nitric oxide (NO) production of macrophage, the activity of T cells and B cells [6, 7]. Therefore, to improve immunity, many research groups have tried to develop immunomodulatory agents from traditional Oriental medicine.

*Cordyceps militaris* (family *Clavicipitaceae, C. militaris*) is a widely used traditional oriental medicine in East Asia to treat immune-dysregulated diseases such as cancer. Previous studies have reported that *C. militaris* extracts possess immune boosting properties, anti-inflammatory activities [8, 9], and anti-cancer activities against lymphoma [10], leukemia [11], bladder cancer [12], breast cancer and colorectal cancer [13]. However, it is difficult to obtain *C. militaris* in large amounts from nature owing to its rarity. Instead of the dead bodies of insects, our research group cultivated *C. militaris* using germinated *rhynchosia nulubilis* (GRC) as a culture media because they contained bioactive components. Previously, our group demonstrated that an extract prepared from *C. militaris* grown on germinated soybeans (GSC) exhibited various biological activities, including anti-allergic activities in vivo and in vitro [14, 15]. Additionally, acidic polysaccharides from GSC exerted anti-viral activity and induced macrophage phagocytic activity [16]. However, GRC is not efficient to commercially utilize due to high cost and low extraction yield after the extraction procedure. As traditional fermented food is known to exert many health effects, researchers have been trying to prove the biological properties of natural products fermented using lactic acid bacteria (LAB). LAB are gram-positive organisms found in vegetables, meats, dairy products, and many parts of the human body, including the gastrointestinal tract. Probiotics can be defined as live microbial food ingredients that exert anti-mutagenic, anti-carcinogenic, and anti-inflammatory activities, reduce serum cholesterol, and modulate immune responses [17]. In addition, fermentation by LAB can enhance preservation duration, taste, flavor, texture, as well as augmenting physiological activities such as digestion efficiency and natural substance metabolism [18]. Here, GRC fermented using different probiotic strains can be used without expensive extraction procedure and exert enhanced biological activities, compared to GRC.

To the best of our knowledge, no studies have analyzed the effects of GRC-ON89A on macrophage activity and immune function in normal and CY-induced immunosuppressed mice. The main objective of this study was to investigate whether GRC fermented with *Pediococcus pentosaceus* has improved immunostimulatory activities compared to unfermented GRC. This is the first report demonstrating the immunostimulatory effects of GRC-ON89A in both healthy and immunosuppressed mice.

## Methods

### Chemicals and reagents

DMEM, fetal bovine serum (FBS) (Gibco/invitrogen, Carlsbad, CA, USA), a Cell Counting Kit-8 assay (Dojindo Lab, Kumamoto, Japan), β-glucan kit (Megazyme International, Wicklow, Ireland), Phagocytosis Assay Kit (Cayman, MI, USA), cyclophosphamide (Sigma Aldrich, St.Louis, MO, USA) β-1,3-glucan (Sigma Aldrich, St.Louis, MO, USA) and cordycepin (Sigma Aldrich, St.Louis, MO, USA) were purchased. Phospho-Lyn antibody, Lyn antibody, phospho-Syk antibody, Syk antibody, phospho-ERK antibody, ERK antibody, phospho-p38 antibody, p38 antibody, phospho-JNK antibody, JNK antibody, NFκB antibody, phospho-IκB antibody and IκB antibody were obtained from Cell signaling Technology Inc. (Danvers, MA, USA). β-Actin antibody was obtained from Santa Cruz (Dallas, TX, USA).

### Preparation of GRC fermented with probiotic strains

The extract of *C. militaris* grown on GRC is prepared using patented technologies developed by Cell Activation Research Institution (CARI, Seoul, Korea), where a voucher specimen was deposited (Kucari: 0903). *Pediococcus pentosaceus* ON89A isolated from onion, *Lactobacillus pentosus* SC64 isolated from pickled burdock, and *Weissella cibaria* Sal.Cla22 isolated from salted clam strains used in this study were obtained from Dr. YS Park. GRC (5% *w*/*v*) was extracted with distilled water (D.W) at 105 °C for 2 h and then cultured with the indicated probiotic strains for 24 h. GRC-inoculated probiotics were heat-killed at 100 °C for 10 min and sonicated for 3 min (Sonics & Materials, Inc., WA, USA) [19].

### Cell cultures

The RAW 264.7 murine macrophage cell line was obtained from the American Type Culture Collection (ATCC, Manassas, VA, USA) and cultured in DMEM with 100 U/ml penicillin and streptomycin. The cells were grown in a 100 mm dish culture at 37 °C with 5% CO_2_ in a humidified atmospheric pressure chamber.

### Quantification of nitric oxide (NO)

Both RAW 264.7 cells and primary cultured peritoneal macrophages were seeded in 96-well plates (2 × 10^5^ cells/well) and then stimulated with various concentrations of the indicated samples (GRC-SC64 (voucher specimen number Gaucari: 0802), GRC-ON89A (voucher specimen number Gaucari: 0801), Sal.Cla22 (voucher specimen number Gaucari: 0803)). After 48 h, the culture supernatants were collected, and NO concentrations were measured using griess reagent. Equal volumes of Griess reagent (1% sulfanilamide, 0.1% naphthylenediamine dihydrochloride, and 0.5% phosphoric acid) and the sample were incubated together at room temperature for 10 min. The absorbance at 540 nm was measured using a microplate reader (Epoch, Biotek Instruments, INC., VT, USA) as described previously [20].

### Cell proliferation assay

RAW264.7 cell proliferation was measured using the Cell Counting Kit-8 (CCK-8) assay (DOJINDO Laboratories, Kumamoto, Japan). RAW264.7 cells were plated onto 96-well plate (1 × 10^4^ cells/well) and then treated with indicated samples (SC64, ON89A, sal.cla22) for 48 h. Peritoneal macrophages were harvested from GRC-ON89A-treated BALB/c mice as described previously [21] and were resuspended in RPMI 1640 (containing 10% FBS) media, then plated into 96-well culture plates (2 × 10^5^ cells/well). They were incubated with a CCK-8, solution for 4 h at 37 °C under 5% CO_2_. After adding CCK-8 solutions, cell proliferation was measured using a microplate reader at 450 nm (Epoch, Biotek Instruments, INC, Winooski, VT, USA). Data were expressed as a percentage relative to proliferation of control cells.

### Animals

Female 6- to 8-week-old BALB/c mice with body weights of 18 ± 2 g were obtained from Daehanbiolink Co., Ltd. (Seoul, Korea). The animals were acclimatized to laboratory conditions for 7 days prior to the commencement of experimentation. The mice were maintained under constant conditions (temperature: 22 ± 2 °C, humidity: 40–60%, light/dark cycle: 12 h) and allowed free access to water and food. All of the animal experiments were performed in accordance to the instructions of the Medical Ethics Committee for the Use of Experimental Animals at Gachon University (GIACUC-R2015014).

### Experimental design

Animal experiments were performed in both normal and immunosuppressed murine models. For animal experiments using normal mice, mice were randomly divided into three groups (> 10 mice per group): normal control group (Control), GRC-ON89A-treated groups (GRC-ON89A) and GRC-treated groups (GRC). For animal experiments using immunosuppressed mice, mice were randomly dived into three groups (> 10 mice per group): CY-injected control (Control-CY), CY-injected and GRC-ON89A-treated groups (GRC-ON89A-CY), and CY-injected and GRC-treated groups (GRC-CY). Immunosuppression was induced by intraperitoneal injection of CY (80 mg/kg) once per day for the initial and final 3 consecutive days. From day 1 to 10 or 19, the four different groups of mice were orally administered D.W, GRC-ON89A, or GRC daily at doses of heat-inactivated 1.0 × 10^8^ CFU/ml/mouse. Twenty-four hours after the final administration, mice were weighed and sacrificed. Mice were euthanized by injecting 1.5% tribromoethanol in tert-amyl alcohol intraperitoneally (Avertin, Sigma Aldrich, MO, USA). The spleen and thymus were immediately removed and weighed. A schematic of the experimental design is shown in Fig. 3a and Fig. 4a. The splenic and thymic indices were calculated as the spleen and thymus weight/body weight, respectively.

### Isolation of primary cultured peritoneal macrophages

Peritoneal macrophages were obtained according to previously described methods [21]. Briefly, the mice were sacrificed to harvested peritoneal macrophages. The mice were sacrificed and 5 mL of PBS was injected intraperitoneally. After massaging the abdomen gently for 3 min, the peritoneal fluid was extracted and centrifuged at 1500 rpm for 10 min. The cell pellets were then suspended in RPMI1640 (supplemented with 10% FBS, 100 U/ml penicillin and 100 U/ml streptomycin), seeded in plates at a density of 2 × 10^5^ cells/ml, and allowed to adhere for 4 h at 37 °C in a humidified incubator containing 5% CO_2_. After 4 h incubation, non-adherent cells were removed by washing twice with PBS and freshly prepared medium was added.

### Phagocytic activity of peritoneal macrophages and RAW 264.7 cells

Isolated peritoneal macrophages (5 × 10^4^ cells per well) and RAW 264.7 cells (2 × 10^5^ cells per well) were plated in 96-well plates and incubated for 24 h in a humidified atmosphere with 5% CO_2_ at 37 °C. GRC and GRC-ON89A were treated for 24-48 h. Phagocytosis activity was evaluated using Cayman Phagocytosis Assay Kit (Cayman, MI, USA). Following manufacturer’s instructions, latex bead-rabbit IgG-FITC complexes were added directly to pre-warmed culture medium to a final dilution of 200:1. The images (magnification, 100×) were obtained every 2 h for 48 h (Nikon ECLIPSE Ti, Tokyo, Japan). The degree of phagocytic activity was measured by analyzing obtained images using MetaMorph software version 7.8.9.0 (Molecular Devices, Sunnyvale, USA). Image analysis was performed using the NIH Image J software (Wright Cell Imaging Facility) and spot counting was done to quantify the number of phagocytized particle signals per cell [22].

### RNA isolation, cDNA synthesis, and reverse transcription-polymerase chain reaction (RT-PCR)

RT-PCR was performed as previously described [23]. RNA was extracted using TRIzol Reagent (Life Technologies, Washington, USA). The concentration and quality of the RNA was detected using Take3 Micro-Volume Plates (Epoch, Biotek Instruments, INC, VT, USA). cDNA synthesis reactions and RT-PCR were performed according to the manufacturer’s instructions (First Strand cDNA Synthesis Kit, Vilnius, Lithuania) and normalized against the expression of glyceraldehyde 3-phosphate dehydrogenase (GAPDH) as a housekeeping gene. Total RNA was isolated from serum, tissue, and cultured RAW 264.7 cells. The following primers were used in this study: GAPDH, 5′-GCA AAG TGG AGA TTG TTG CCA TC-3′ (forward) and 5′-CAT ATT TCT CGT GGT TCA CAC CC-3′ (reverse); TNF-α 5′-ATG AGC ACA GAA AGC ATG ATC CG-3′ (forward) and 5′-CCA AAG TAG ACC TGC CCG GAC TC-3′ (reverse); IL-10 5′-TGC TAT GCT GCC TGC TCT TA-3′ (forward) and 5′-GGC AAC CCA AGT AAC CCT TA-3′ (reverse). The PCR program for GAPDH was as follows: initial denaturation at 94 °C for 2 min, followed by 30 cycles of denaturation at 94 °C for 20 s, annealing at 58.4 °C for 10 s, and extension at 72 °C for 30 s, with a final extension at 72 °C for 5 min. The PCR program for TNF-α was as follows: initial denaturation at 94 °C for 2 min, followed by 30 cycles of denaturation at 94 °C for 20 s, annealing at 60 °C for 10 s, and extension at 72 °C for 45 s, with a final extension at 72 °C for 5 min. The PCR program for IL-10 was as follows: initial denaturation at 94 °C for 2 min, followed by 30 cycles of denaturation at 94 °C for 20 s, annealing at 55.4 °C for 10 s, and extension at 72 °C for 40 s, with a final extension at 72 °C for 5 min.

### Western blotting analysis

Western blotting analysis was performed as described previously [20]. Protein concentrations were determined using Pierce BCA (Bicinchoninate) Protein Assay Kit (Thermo SCIENTIFIC, Meridian, Rockford, USA). Total protein was separated using SDS PAGE on a 10% polyacrylamide gel, followed by electrophoretic transfer onto nitrocellulose membranes (Bio-Rad Laboratories, Inc., Hercules, CA, USA). The membranes were incubated with 5% skim milk solution, followed by incubation with a primary antibody. The membranes were washed in a 1 x TBST (20 mM Tris, 500 mM sodium chloride, pH 7.6, and 0.1% Tween 20) buffer and incubated with horseradish peroxidase-conjugated secondary antibodies (Santa Cruz, CA, USA) for 1–2 h. The immunoreactive bands were detected using the enhanced chemi-luminescence western blotting detection system by odyssey LCI Image software (LI-COR Biosciences, Lincoln, NE, USA).

### β-glucan content

The β-glucan content in GRC and GRC-ON89A was determined using mushroom and yeast specific β-glucan kit as the manufacturer’s protocols. The absorbance of all solutions was analyzed spectro-photometrically at 510 nm microplate reader (Epoch, Biotek Instruments, INC., VT, USA). The β-glucan content was calculated by subtracting the α-glucan content from the total glucan content. These calculations can be simplified by using the Megazyme Mega-Calc™. The β-glucan content was expressed as g/100 g of mushroom dry weight.

### The quantitative analysis of cordycepin in GRC-ON89A

The content of cordycepin was determined by UPLC quantitative analysis of GRC-ON89A, as the previously described methods [24]. The UPLC-Q-TOF-MS analysis were performed on a Waters Micromass Q-TOF Premier with a UPLC Acquity system (Waters Corp., Milford, MA, USA) equipped with a binary solvent delivery system, an auto-sampler, and UV (Ultraviolet Ray) detector. The separation was performed on an Acquity UPLC BEH C18 (100 mm × 2.1 mm × 1.7 μm particle size; Waters Corp.). UPLC-Q-TOF-MS analysis condition were as follows: injection volume 5 μl, flow rate 0.3 m/min, column temperature 37 °C. The mobile phases consisted of 0.1% formic acid in water (solvent A) and 0.1% formic acid in ACN (solvent B); the initial condition was 5% B for 1 min, then B was gradually increased to 100% during 10 min; the 100% B was maintained for 1 min and then decreased to 5% B during 3 min. The total run time was 14 min.

### Short chain fatty acid (SCFA) analysis

SCFA was analyzed by Gas chromatography/Mass spectrometer (GC/MS, Agilent 7890B, Agilent Technologies, Inc., Santa Clara, CA, USA) at the Korea Basic Science Institute (Seoul, Korea, Western Seoul Center) as previously described [25].

### Statistical analysis

Data are expressed as means ± SEM or means ± SD. Differences between groups were determined by one-way ANOVA (Analysis of variance) followed by Dunnett’s t-test or Student’s t-test. Statistical analysis was performed using SPSS, version 12 (SPSS Inc., Chicago, IL, USA).

## Results

### Screening GRC deposited at the ted by candidate probiotics for immune stimulatory activity

**Fig. 1:**
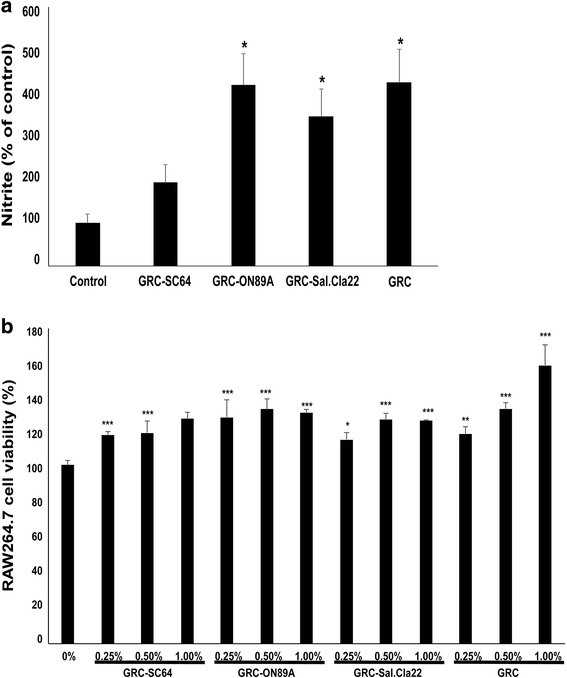
Effects of GRC fermented with different probiotic stains on nitric oxide (NO) production. **a** RAW 264.7 cells were treated with GRC-SC64, GRC-ON89A, GRC-Sal.Cla22, or GRC. Nitrite levels in the culture media were determined using Griess reagent and were presumed to reflect NO levels. **b** RAW 264.7 cells were treated with various concentrations (0.25%, 0.50%, and 1.00%) of SC64, ON89A, Sal.Cla22, or GRC for 24 h. RAW 264.7 cell proliferation was assessed using a CCK-8 assay. One-way ANOVA was used for comparison of group means, followed by Dunnett’s *t*-test (* = *p* < 0.05, ** = *p* < 0.01, *** = *p* < 0.005 vs. control group). Each figure is representative of three independent experiments

NO, released by macrophages, plays an important role in the innate immune response. NO levels are indirectly calculated by measuring the level of nitrites and nitrates. Among GRC extracts fermented with different probiotic strains, we screened out the one with the strongest immunostimulatory activity by measuring the level of NO produced by treated RAW 264.7 macrophages. The NO level released from GRC fermented with various probiotic stains (SC64, ON89A, Sal.Cla22) were compared with GRC alone. Among these, GRC-ON89A significantly increased NO production in RAW264.7 cells (Fig. 1a). The potential cytotoxicity of these samples on RAW 264.7 macrophages was investigated by measuring cell viability using a CCK-8 assay. All samples significantly increased RAW 264.7 cell viability (Fig. 1b). Therefore, we chose GRC-ON89A as the test extract for future immunoregulatory studies.

### GRC-ON89A increases phagocytic activity in RAW 264.7 and primary peritoneal macrophages

**Fig. 2:**
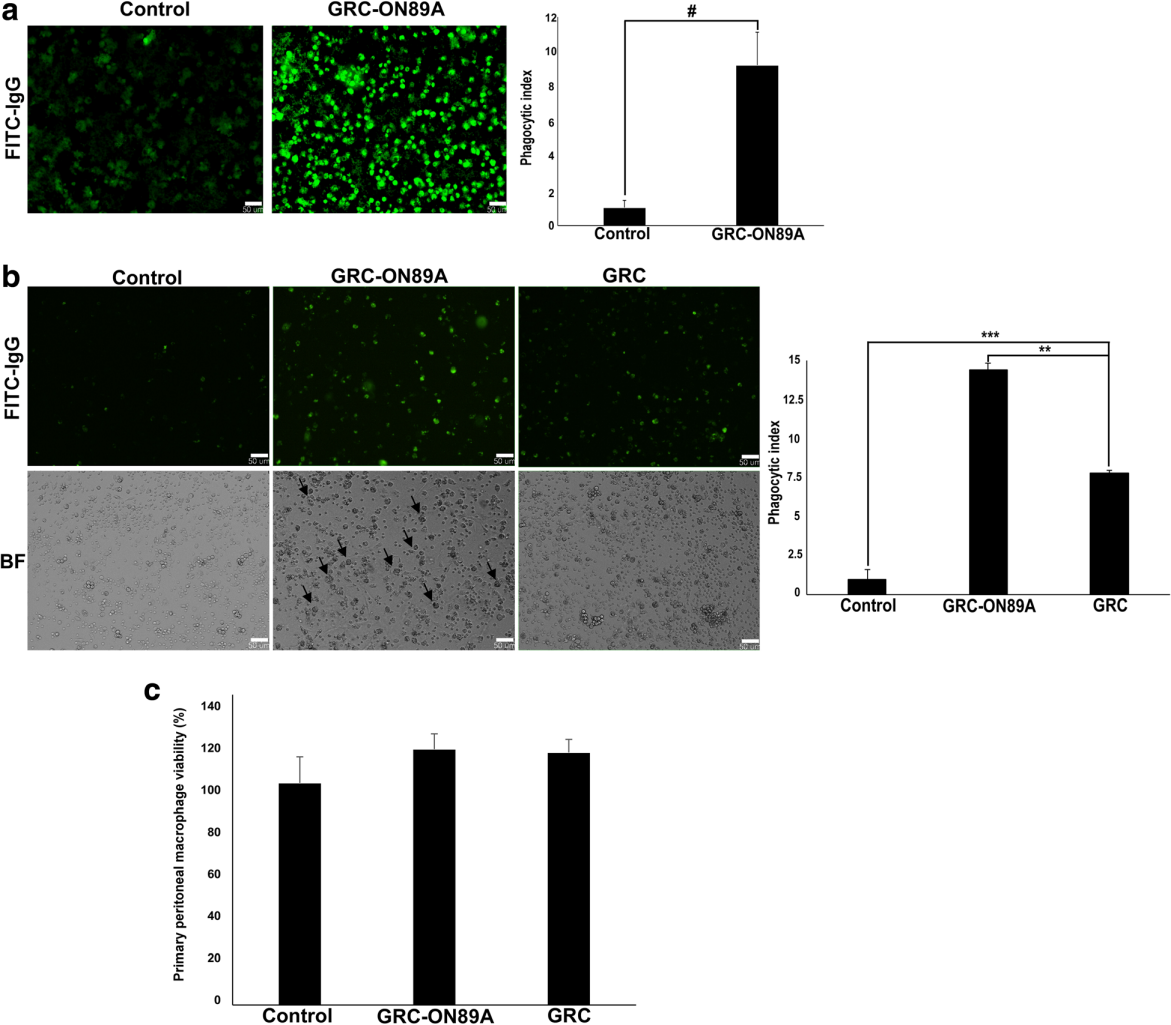
Effects of GRC-ON89A on phagocytic activity of RAW 264.7 cells and primary cultured peritoneal macrophages. The phagocytosis activity of 1% GRC-ON89A-treated macrophages was examined in the presence of IgG-opsonized FITC particles for 48 h. **a** The phagocytic activity of RAW 264.7 macrophages and (**b**) primary peritoneal macrophages. Real-time cell images were taken and analyzed using Metamorph software (magnification, 100×). The phagocytic index was quantified for statistics analysis using Image J software. Data comparisons among groups were analyzed using Student’s *t*-test and One-way ANOVA with Dunnett’s *t*-test for significance of individual comparisons (# = *p* < 0.05 vs. non-treated control group, ** = *p* < 0.01, *** = *p* < 0.001 vs. GRC). **c** Primary peritoneal macrophag e viability was assessed by CCK-8 assay. Data are represented as the mean ± SD (*n* > 3) or ± SEM (*n* > 10). BF denotes bright field. Each figure is representative of three independent experiments; Scale bars = 50 μm

We investigated whether GRC-ON89A affected the phagocytic activity of RAW 264.7 macrophages and primary cultured peritoneal macrophages. GRC-ON89A significantly increased the phagocytic activity of RAW 264.7 cells (Fig. 2a) and primary cultured peritoneal macrophages (Fig. 2b) compared to non-treated control (* = *p* < 0.5, *n* > 10) or GRC (** = *p* < 0.01, *n* > 10). GRC-ON89A-treated primary cultured peritoneal macrophages increased in size, compared to control and GRC-treated primary cultured peritoneal macrophages. This morphological change suggests that GRC-ON89A may induce primary peritoneal macrophage activity (Fig. 2b). No cytotoxic effects of GRC-ON89A were observed on primary peritoneal macrophages (Fig. 2c). These data demonstrate that GRC-ON89A was a stronger stimulator of phagocytic activity than GRC.

### Effects of GRC-ON89A on phagocytic activity of peritoneal macrophages from CY-treated immunosuppressed mice and normal mice

**Fig. 3:**
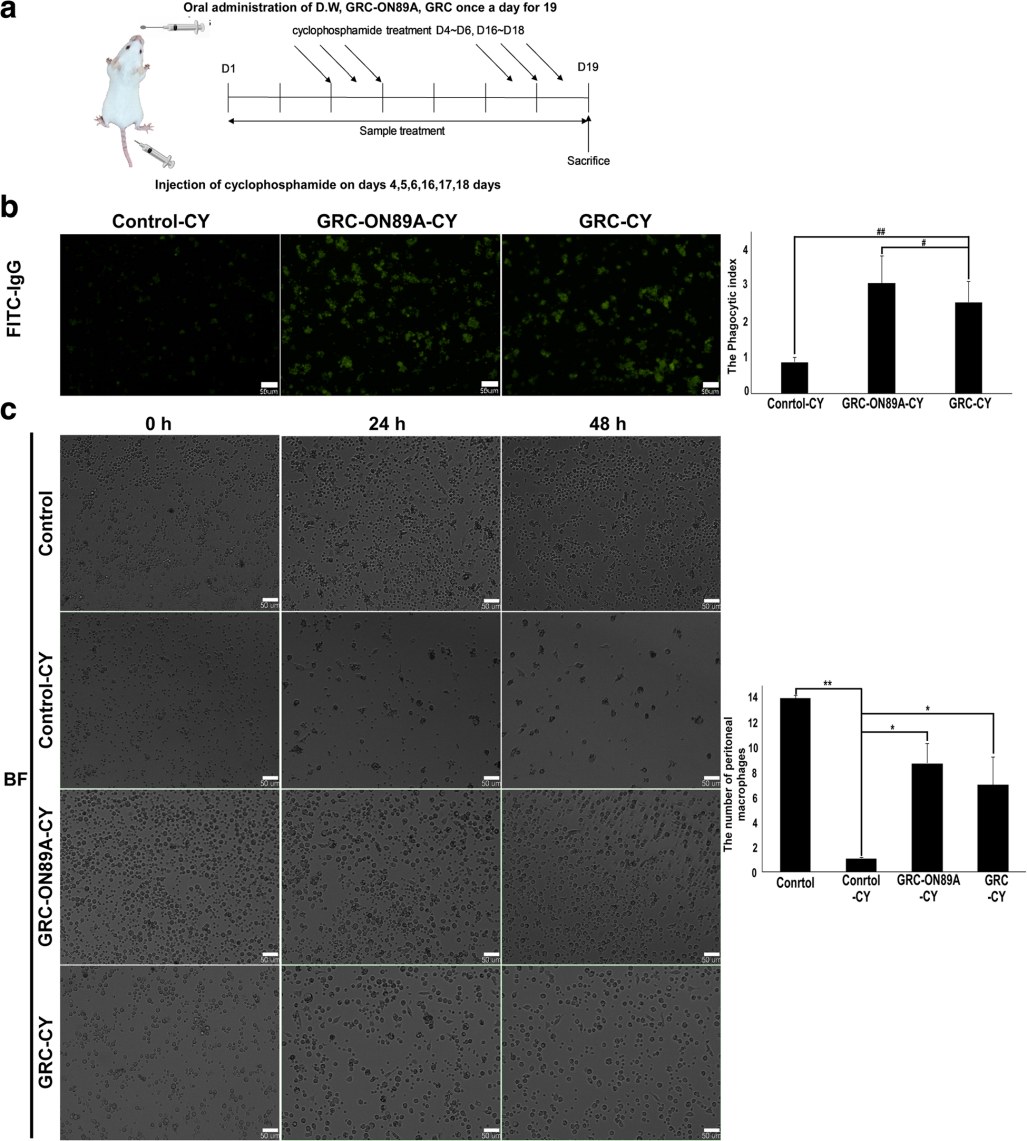
Effects of GRC-ON89A on the phagocytic activity of peritoneal macrophages in CY-treated mice. **a** Scheme summarizing the immunosuppressed murine model protocol. **b** Fluorescent microscopy images (left panel) of phagocytosis of primary peritoneal macrophages from CY-treated immunosuppressed groups. Cells (5 × 10^4^ cells per well) were treated with DMSO vehicle (100μg/mL GRC and 100μg/mL GRC-ON89A). Quantification of fluorescent bead uptake by primary peritoneal macrophages from CY-treated immunosuppressed groups exposure to fluorescent beads for 24 h (right panel). One-way ANOVA was used for comparison of group means, followed by Dunnett’s *t*-test (## = *p* < 0.01, # = *p* < 0.05 vs GRC-CY group). **c** Effect of GRC-ON89A on primary peritoneal macrophage proliferation. The cell image (left panel) of primary peritoneal macrophages from CY-treated immunosuppressed model group after oral administration (D.W., 1% GRC and 1% GRC-ON89A). The cell proliferation rate was quantified for statistics analysis using Image J software (right panel). One-way ANOVA was used for comparison of group means, followed by Dunnett’s *t*-test (** = *p* < 0.01, * = *p* < 0.05 vs control-CY group). Each figure is representative of three independent experiments; Scale bars = 50 μm

**Fig. 4:**
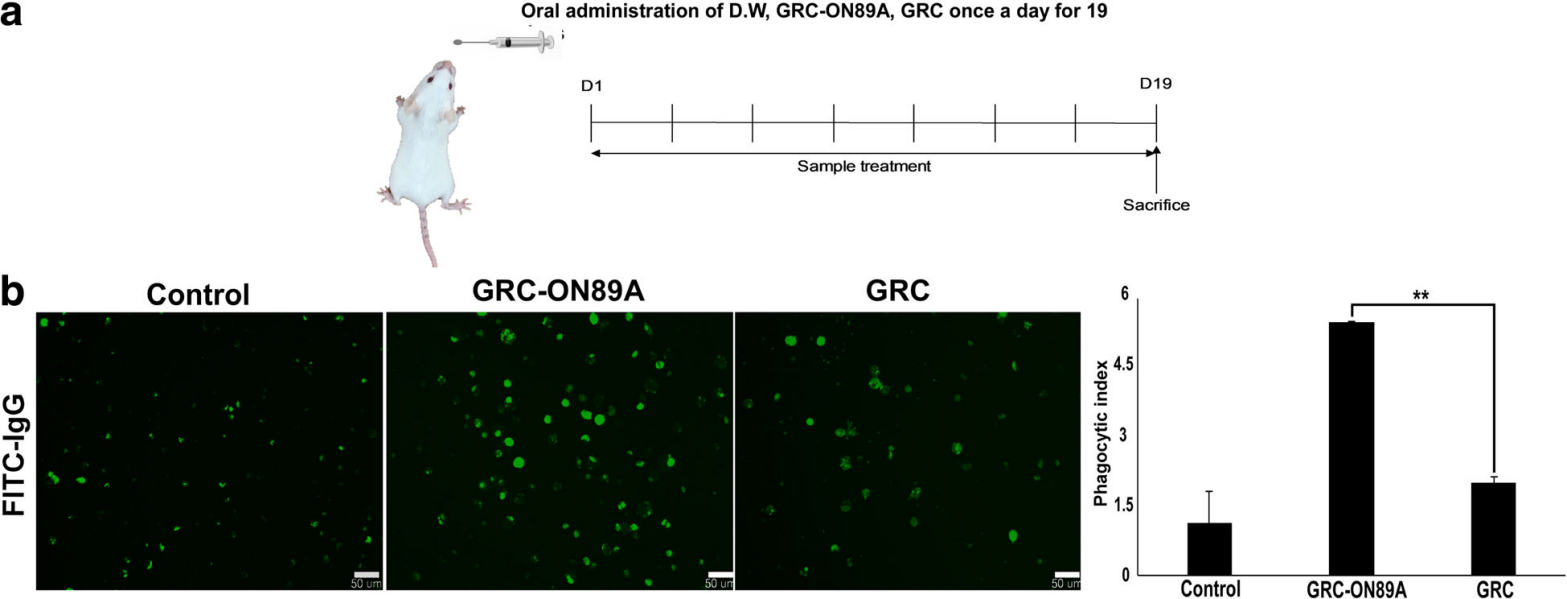
Effects of GRC-ON89A on the phagocytic activity of peritoneal macrophages in normal mice. **a** Scheme summarizing the normal murine model protocol. **b** Fluorescent microscopy images (left panel) of phagocytosis of primary peritoneal macrophages from CY-treated immunosuppressed model group after oral administration (D.W., 1% GRC and 1% GRC-ON89A). Quantification of fluorescent bead uptake by primary peritoneal macrophages from CY-treated immunosuppressed groups exposure to fluorescent beads for 48 h (right panel). The phagocytic index was quantified for statistics analysis using Image J software. One-way ANOVA was used for comparison of group means, followed by Dunnett’s *t*-test (** = *p* < 0.01 vs GRC). Each figure is representative of three independent experiments; Scale bars = 50 μm

The phagocytic activity of peritoneal macrophages from GRC-ON89A was assessed using IgG-opsonized FITC particles in immunosuppressed (Fig.3a) and normal (Fig.4a) murine models. Macrophage phagocytic activity decreased significantly after CY treatment. As shown Fig. 3b, GRC-ON89A significantly increased the phagocytic activity of peritoneal macrophages from CY-treated mice compared to GRC (* = *p* < 0.05). However, there was no significance in the phagocytic activity of peritoneal macrophages from mice after oral administration of GRC-ON89A and GRC since the standard deviation among mice was too big (data not shown). In the CY-treated immunosuppressed model, the number of peritoneal macrophages in the GRC-ON89A administered group was found to be significantly increased compared to CY-treated and GRC-treated groups (Fig. 3c). Additionally, we observed that the phagocytic rate of peritoneal macrophages from normal mice after oral administration of GRC-ON89A significantly increased compared to those from GRC (** = *p* < 0.01, Fig. 4b).

### Effects of GRC-ON89A on the phagocytosis related signaling molecules and on the levels of cytokine mRNA expression

**Fig. 5:**
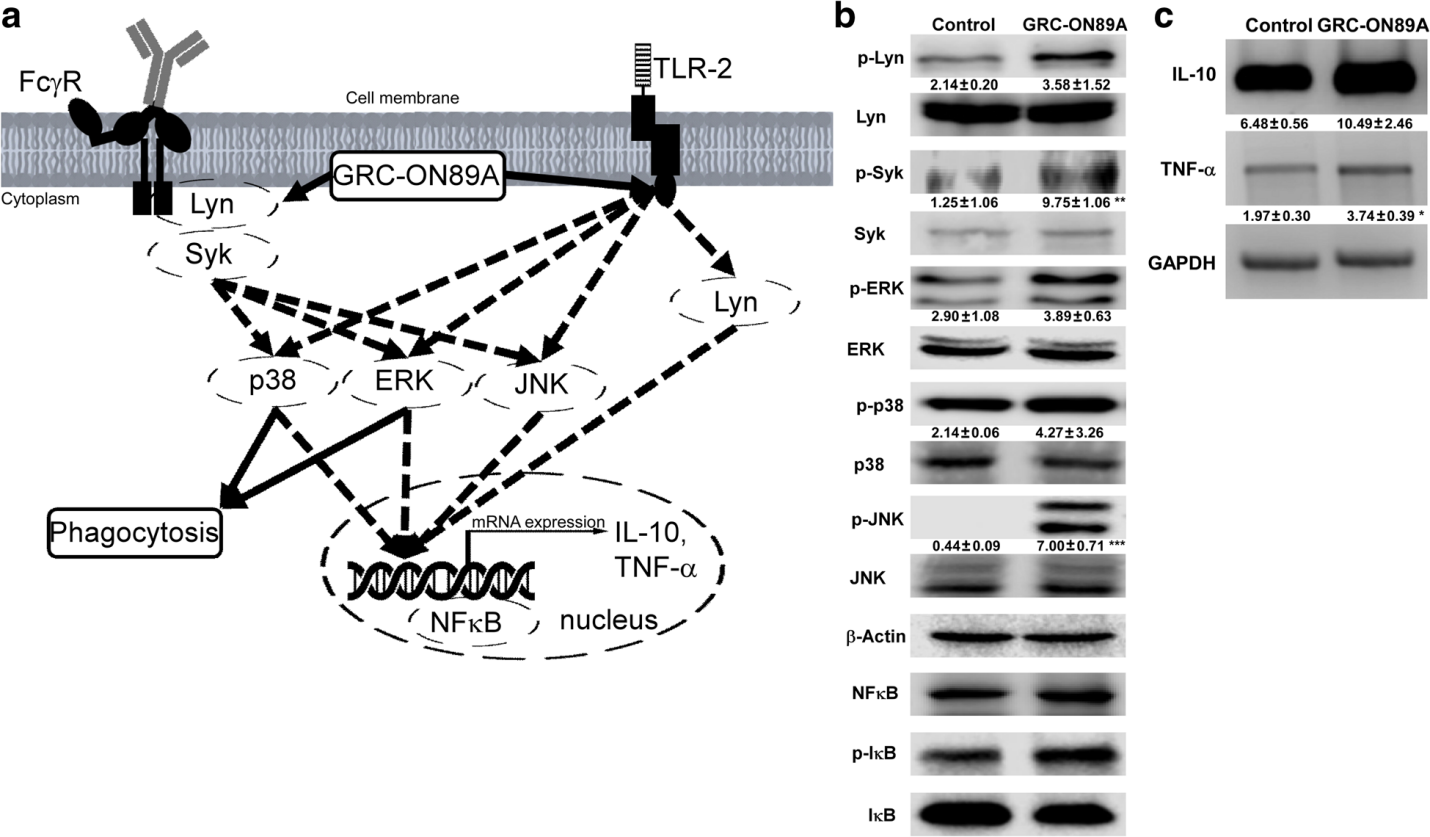
The effect of GRC-ON89A on the phagocytosis related signaling molecules and on the levels of cytokine mRNA expression. **a** GRC-ON89A activated FcγR signaling pathway. RAW264.7 cells (1 × 10^6^ cells/ml) were treated with GRC-ON89A for 1 h. **b** Total protein lysates were analyzed by Western blot analysis for phosphorylated – and total forms of Lyn, Syk, ERK, p38, JNK, NFκB, and IκB. β-Actin was used as the internal control. **c** The expression level of IL-10 and TNF-α mRNA in RAW 264.7 cells was determined by RT-PCR. Each figure is representative of three independent experiments. Data comparisons between groups were analyzed using Student’s *t*-test and One-way ANOVA with Dunnett’s *t*-test for significance of individual comparisons (* = *p* < 0.05, ** = *p* < 0.01, *** = *p* < 0.001 vs. control group)

To determine whether GRC-ON89A affects signaling pathways, we examined the level of phosphorylation in Lyn, Syk, and MAPKs. We observed increased phosphorylation of Lyn, Syk, and MAPKs after GRC-ON89A treatment in RAW264.7 cells (** = *p* < 0.01, *** = *p* < 0.001 vs non-treated control, Fig.5b). We observed nuclear translocation of NFκB and phosphorylation of IκB protein were increased and IκB protein was decreased. These results suggest that GRC-ON89A induces phagocytic activities by upregulating the activation of Lyn, Syk, MAPKs, and nuclear translocation of NFκB. Figure 5a illustrates a model detailing the role of GRC-ON89A in the phagocytic signaling pathways. GRC-ON89A treatment induced significantly higher TNF-α and IL-10 mRNA expression in RAW 264.7 cells (* = *p* < 0.05 vs non-treated control, Fig. 5c).

### Splenic and thymic indices of GRC-ON89A in normal and immunosuppressed mice

As shown in Table 3a, we found that splenic and thymic indices slightly increased in GRC-ON89A-treated groups compared to vehicle-treated and GRC-treated groups. In immunosuppressed mice (Table 3b), splenic and thymic indices slightly increased in GRC-ON89A-treated groups compared to vehicle-treated groups. (** = *p* < 0.01 vs CY treated group).

### The content of bioactive compounds in GRC before and after fermentation with *P. pentosaceus* (ON89A)

**Fig. 6:**
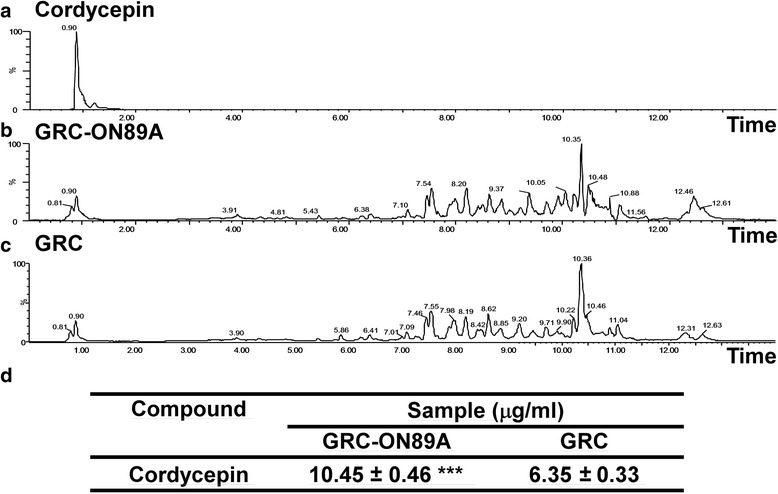
Characterization of cordycepin content by UPLC-Q-TOF-MS. UPLC-MS chromatograms of (**a**) cordycepin, (**b**) GRC-ON89A and (**c**) GRC. **d** The quantitative analysis of cordycepin content in GRC before and after fermentation with ON89A. (*** = *p* < 0.005 vs. GRC, *n* > 3)

To elucidate the enhanced immune activity of GRC-ON89A, we compared the content of several compounds in GRC before and after fermentation with ON89A. Our data indicated that β-glucan content of GRC and GRC-ON89A was 11.81 ± 0.35 g/100 g and 19.26 ± 0.61 g/100 g, respectively (Table 1). The content of β-glucan in GRC-ON89A has higher than that in GRC. This result that ON89A might break down the high molecular weight of β-glucan from GRC. Next, we measured the content of cordycepin, known as a major component of *C.miilitaris*. A HPLC profile, including standard sample and GRC-ON89A, is shown in Fig. 6. The concentration of cordycepin from GRC-ON89A and GRC was 6.35 ± 0.33 μg/mg and 10.44 ± 0.46 μg/mg, respectively (Fig. 6d). The content of cordycepin was increased in GRC-ON89A, compared to that in GRC. Our results are in agreement with other studies that an increase in acetic acid, propionic acid and lactic acid in GRC after ON89A fermentation. (Table 2). Our data showed that GRC-ON89A increased bioactive compounds compared to GRC. This result is consistent with the enhanced immune stimulatory activity in GRC-ON89A treated group, compared to GRC treated group.

**Table 1:** β-glucan content in the GRC-ON89A and GRC

Sample	Total glucan	α-glucan	β-glucan
GRC-ON89A	24.64 ± 0.91^A^	5.38 ± 0.30^A^	19.26 ± 0.61^B^
GRC	19.67 ± 0.24	7.86 ± 0.14	11.81 ± 0.35

All values are expressed as mean ± SD. of triplicate measurements (^A^ = *p* < 0.05, ^B^ = *p* < 0.005 vs. GRC, *n* > 3)

**Table 2:** The SCFA (acetic acid, propionic acid, lactic acid) content in GRC-ON89A and GRC

Compound	Sample (μg/ml)	
	GRC-ON89A	GRC
Acetic acid	665	508
Propionic acid	1.9	0.2
Lactic acid	9155.5	8795.5

### The effect of bioactive compound in GRC-ON89A on macrophage activity

**Fig. 7:**
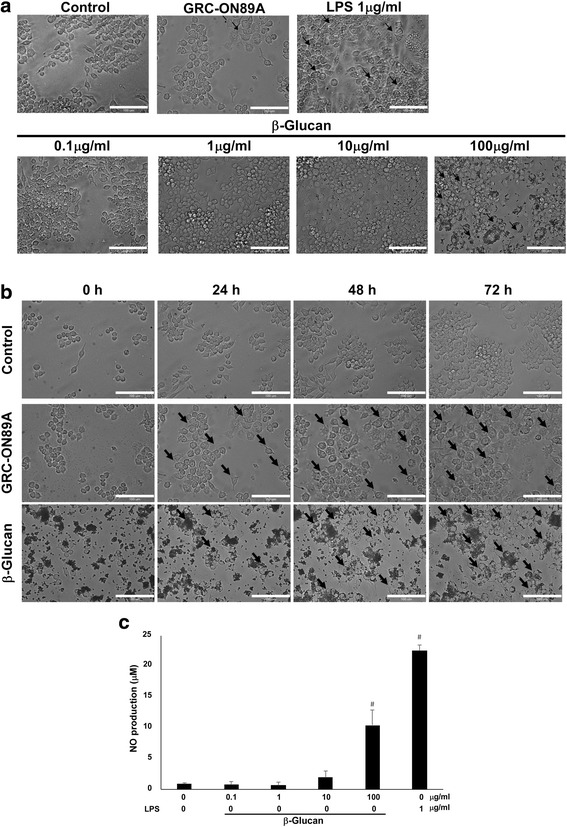
Effect of GRC-ON89A and β-glucan on macrophage activity and morphology. **a** Cell morphological changes of β-glucan or GRC-ON89A treated RAW 264.7 cells. RAW 264.7 cells were treated with GRC-ON89A, β-glucan (0.1, 1, 10, 100 μg/ml) or LPS (1 μg/ml). Arrows mediated RAW264.7 cells with increased cellular size. **b** The cell morphology of RAW 264.7 by GRC-ON89A, β-glucan (100 μg/ml) for 0, 24, 48, and 72 h was observed using Metamorph software for real time imaging (magnification, 200×). Arrows mediated RAW 264.7 cells with increased cellular size. **c** The level of NO production in RAW 264.7 cells by β-glucan. One-way ANOVA was used for comparison of group means, followed by Dunnett’s *t*-test (^#^ = *p* < 0.005 vs. control group). Each figure is representative of three independent experiments; Scale bars = 100 μm

We checked the effect of β-glucan and GRC-ON89A on macrophages activity. The β-glucan-, GRC-ON89A- or LPS- treated RAW264.7 cells were increased in cellular size and numbers (Fig. 7a and b). In addition, the released amount of NO was increased in β-glucan treated RAW264.7 cells (Fig. 7c). These results suggest that the immuno-stimulatory effects of GRG-ON89A should be closely associated with the characteristic of β-glucan.

## Discussion

The main objective of this study was to investigate the immunostimulatory effects of GRC fermented with *Pediococcus pentosaceus*. There is growing interest in identifying plant- or probiotic-originated immunomodulators for prophylactic and therapeutic purposes [26]. The general most commonly used in probiotic applications are *Lactobacillus*, *Pediococcus*, *Weissella*, *Streptococcus*, and *Bifidobacterium*. GRC fermentation differs with regard to various probiotic strains and was evaluated for its impact on immune regulatory activities. In this study, we demonstrated the immunostimulatory activities of GRC fermented with ON89A, including its macrophage-activating and immune function-enhancing properties in a normal mouse model. CY is often used in chemotherapy and organ transplantation [27]. One of main side effects of CY treatment is an immunosuppressive effect caused by damaged DNA in normal cells. Thereby, we also investigated the protective effects of GRC-ON89A in a CY-induced immunosuppressed mouse model. Macrophages exist in almost all tissues and regulate both innate and adaptive immunity [28]. They are derived from peripheral blood monocytes and present antigens to other immune cells via phagocytic activity. Activated macrophages produce great amounts of reactive oxygen/nitrogen species, as well as several cytokines, which contribute to the killing of bacteria- or virus-infected cells and tumor cells [29]. To identify the most active probiotic-fermented GRC sample, we examined NO release. Among the investigated samples, GRC-ON89A induced the greatest NO production (Fig. 1a). Thus, the immunological effects of GRC-ON89A were evaluated in this study.

NO is known to regulate the production of cytokines, such as TNF-α, by macrophages and T lymphocytes [30]. Activated macrophages are associated with cytokines such as interferon-γ, IL-1β, IL-6, IL-10 and TNF-α to recruit and activate other cells for the initiation of the adaptive immune response [31, 32] GRC-ON89A has been shown to significantly induce TNF-α and IL-10 production in RAW 264.7 cells (Fig. 5c).

The phagocytic function of macrophages is one of the most important non-specific immune responses against foreign organisms. Because phagocytosis by macrophages is the first immune response to invasive cells, including tumor cells and microorganisms, we investigated whether GRC-ON89A could enhance phagocytic activity. The phagocytic activity was monitored by detecting internalized IgG-opsonized FITC particles in macrophages and higher fluorescence emission intensity indicates increased phagocytosis activity. GRC-ON89A enhanced the phagocytic activity of RAW 264.7 cells (Fig. 2a), and primary peritoneal macrophages from normal mice (Fig. 2b). In addition, we evaluated whether GRC-ON89A could recover weakened immunity. CY suppressed the cell proliferation and the phagocytic activity of macrophages (Fig. 3b and c, left panel), which was consistent with previous studies [33, 34]. GRC-ON89A enhanced the phagocytosis of primary peritoneal macrophages from immunosuppressed (Fig. 3b) and normal mice (Fig. 4b) as measured by the quantification of fluorescent latex bead uptake. The oral administration of GRC-ON89A significantly increased the proliferation of peritoneal macrophages in immunosuppressed mice (Fig. 3c). The present study suggests that GRC-ON89A could enhance non-specific immune function in both immunosuppressed mice and normal mice.

Several studies have shown that Lyn is important in regulating phagocytic activity through recruiting autophagic components to the phagosome [21]. Eradicating Lyn hinders bacteria delivery to lysosomes [35]. Our data reveals that GRC-ON89A induced the phosphorylation of Lyn (Fig. 5). This finding indicated that GRC-ON89A facilitates phagocytic activity through the activation of Lyn, which acts as a link between phagosomes and autophagosomes. The major signaling pathways that lead to enhance phagocytic activities in macrophages include the FcγR pathway, Toll like receptor-2 (TLR-2) pathway, and others. As Src family kinases are important in FcγR signaling, FcγR-mediated phagocytosis is defective in Lyn deficient macrophages in Lyn [36]. Previous studies have found that Syk plays an essential role in phagocytic receptor (i.e. FcγR) signaling [37]. In accordance with previous reports, we observed that phosphorylation of Syk was increased in the GRC-ON89A possesses immune regulatory activity.

Previous studies demonstrated that ERK and p38 MAPK play a key role in macrophage activation and phagocytosis [38, 39]. Activated ERK and p38 MAPK enhance actin polymerization, which is necessary for bacteria internalization by phagocytic activity of macrophages [39, 40]. In addition, it is reported that MAPK activation induces IL-10 production in murine activated macrophages [41]. Furthermore, ERK, p38 MAPK and c-Jun N-terminal kinase (JNK) signaling pathway are activated by FcγRIIa [7]. MAPK and NFκB regulate gene expression of NO and TNF-α that acts on innate immune response [42]. It is reported that NFκB is a role transcription factor in innate immunity [7]. Inhibitory IκB proteins block the nuclear translocation of NFκB dimers. Upon stimulus, IκB kinase (IKK) phosphorylates IκB, which results in its degradation, and release NFκB for nuclear translocation [43]. Activated ERK mediates NFκB activation and also enhanced phagocytosis [44]. In this study, GRC-ON89A increased MAPK phosphorylation and NFκB activation. These results suggest that GRC-ON89A may increase macrophage phagocytic activity through activation of the MAPK and Lyn pathways (Fig. 5). In addition, GRC-ON89A suppressed NO and pro-inflammatory cytokine production in LPS-stimulated macrophages (manuscript in preparation). It is likely that GRC-ON89A regulated both anti-inflammatory and phagocytic activities of macrophages.

Spleen and thymus are the major immune organs where the lymphocytes get matured and differentiated. Among lymphocytes, T cells and B cells are important effector cells in cell-mediated immunity [45]. It is reported that cyclophosphamide suppressed T-lymphocyte proliferation and B-lymphocyte proliferation [46]. When GRC-ON89A was orally administered daily in normal and immunosuppressed murine models, GRC-ON89A treated group showed the increased weight of immune organs, such as the spleen and thymus (Table 3). We plan to study the effect of GRC-ON89A on the lymphocyte function and its mechanism of action in future.

**Table 3:** Effects of GRC-ON89A on immune organs

Group	Number of animals	Splenic index (SI)	Thymic index (TI)
(A)			
Con	10	0.36 ± 0.01	0.30 ± 0.04
GRC-ON89A	10	0.46 ± 0.11	0.35 ± 0.04
GRC	10	0.37 ± 0.02	0.32 ± 0.10
(B)			
Con-CY	10	0.22 ± 0.03	0.13 ± 0.04
GRC-ON89A-CY	10	0.25 ± 0.02 *	0.28 ± 0.04 **
GRC-CY	10	0.27 ± 0.06	0.24 ± 0.05 *

The splenic and thymic indices in (A) normal and (B) immunosuppressed mice. GRC-ON89A was administered daily from day 1 to 19. The control group mice were treated with D.W. The data are presented as the mean ± SEM (*n* > 10). Data were analyzed using the student *t*-test or One-way ANOVA with Dunnett’s *t*-test (** = *p* < 0.01, * = *p* < 0.05 vs control-CY group)

We found there was a change in the content of bioactive compounds in GRC after ON89A fermentation. Several groups reported that (1, 3)-β-D-glucan stimulated macrophage activation [47]. When high molecular weight β-glucan orally administered, it internalized and fragmented in the macrophages after entering the small intestine. These small β-glucan fragments, released from the macrophages, are taken up by other immune cells, which induce variety of immune responses [48]. In this study, we found that the β-glucan content of GRC-ON89A was higher than that of GRC (Table 1). The β-glucan treated RAW264.7 cells increased cell size and NO production (Fig. 7). This suggested that ON89A might have fragmented β-glucan from GRC, not newly producing β-glucan, which might enhance the immune responses in vitro and in vivo. It is reported that SCFA enhances innate immunity as a strong inducer of anti-microbial host defense peptides [49, 50]. Bacterial fermentation of indigestible carbohydrates in the intestine produced the major species of SCFAs, including acetate, propionate, and butyrate [51]. We found that the level of SCFA increased after fermentation with lactic acid bacteria ON89A (Table 2).

Previous studies demonstrated that cordycepin, initially isolated from *Cordyceps*, has many pharmacological activities, including immunostimulatory, anti-cancer and anti-infection activities [52]. Our analytical data indicated the content of cordycepin in GRC increased after fermentation by lactic acid bacteria ON89A (Fig. 6). Altered immune activity of macrophages may be due to the increased content of bioactive materials such as cordycepin in GRC-ON89A. We will persue potential bioactive compounds that are biotransformed after fermentation. In further studies, it would be worth performing the efficacy test through clinical study and immune deficient or cancer-xenograft animal models.

## Conclusions

The results demonstrated that GRC-ON89A could play an ethnopharmacologic role as an immune-stimulant. This study suggests that GRC-ON89A acts as an immune-stimulant in both CY-treated mice and normal mice by enhancing the phagocytic activity of mouse peritoneal macrophages and stimulating NO production in macrophages. It is likely that GRC-ON89A induced the phagocytic activity of macrophages through upregulating TLR-2 and FcγR-mediated signaling, such as tyrosine kinase Syk or other tyrosine-phosphorylated products. GRC after fermentation increased β-glucan, cordycepin and SCFA contents. Our data suggested that GRC-ON89A may be applied as an agent for immune boosting therapy in immune suppressed patients.

## Abbreviations

ATCC: American type culture collection
CCK-8: Cell counting kit-8
CY: Cyclophosphamide
ELISA: Enzyme-linked immunosorbent assay
FBS: Fetal bovine serum
FITC: Fluorescein isothiocyanate
GAPDH: Glyceraldehyde 3-phosphate dehydrogenase
GI: Gastrointestinal
GRC: *C. militaris* germinated *rhynchosia nulubilis*
IkB: Inhibitor of kappa B
IL: Interleukin
LAB: Lactic acid bacteria
LC: Liquid chromatography
Lyn: Lck/yes novel
MAPK: Mitogen-activated protein kinases
MS: Mass spectrometry
NFkB: Nuclear factor kappa-light-chaine-enhancer of activated B cells
NO: Nitric oxide
PBS: Phosphate-buffered saline
PCR: Polymerase chain reaction
RNA: Ribonucleic acid
RT-PCR: Reverse transcription-polymerase chain reaction
SCFA: Short chain fatty acid
Syk: Spleen tyrosine kinase
TNF: Tumor necrosis factor
UPLC: Ultra Performance liquid chromatography
